# In vitro and in vivo evaluations of antioxidative, anti-Alzheimer, antidiabetic and anticancer potentials of hydroponically and soil grown *Lactuca sativa*

**DOI:** 10.1186/s12906-022-03520-5

**Published:** 2022-01-31

**Authors:** Shahid Naseem, Hammad Ismail

**Affiliations:** Department of Biochemistry and Biotechnology, University of Gujrat, Gujrat, 50700 Pakistan

**Keywords:** Anticholinesterase, Antidiabetic, Anti-oxidants, Cytotoxic potential, *Lactuca sativa*, Neurotransmitters, HPLC

## Abstract

**Background:**

*Lactuca sativa* is an edible plant commonly used by local communities to manage diabetes and stomach problems.

**Methods:**

This work aimed to investigate the anti-oxidant, anticancer, antidiabetic and Anti-Alzheimer effects of hydroponically (HyL) and soil-grown (SoL) *Lactuca sativa*. Streptozotocin-induced diabetes and AlCl_3_-induced Alzheimer’s disease model was used to evaluate the medicinal effects of *Lactuca sativa*.

**Results:**

HyL showed significant activity in lipid peroxidation assay, DPPH and DNA protection assay, while SoL extract showed moderated activity, respectively. A similar activity response was quantified for α-glucosidase, α-amylase, acetylcholinesterase and butyrylcholinesterase inhibition assays. The cytotoxic potential of HyL and SoL extracts against MCF7, and HePG2 cancer cell lines exhibited significant activity. HyL and SoL showed a substantial decrease in blood glucose levels in streptozotocin-induced diabetic rats. Diabetes-related liver/kidney biomarkers and anti-oxidant enzyme trends moved toward normal after HyL and SoL treatment. In Anti-Alzheimer’s based Morris water and elevated plus maze tests, HyL and SoL displayed memory-enhancing response and anti-anxiety behaviour, respectively. HPLC quantification of dopamine and serotonin revealed a moderate but significant (*p*<0.05) increase in the level of these neurotransmitters in HyL and SoL groups.

**Conclusion:**

Overall, the study revealed that hydroponic *Lactuca sativa* possesses the therapeutic potential to treat diseases like Alzheimer’s and diabetes.

## Introduction

Diabetes, known as a metabolic disorder, is the insensitivity or deficiency of insulin [1]. Prolonged diabetic impairments may lead to secondary complications. Clinical studies have reported association neurodegeneration like Alzheimer’s disease with diabetes [2, 3]. It is proposed that diabetes induces changes in glucose metabolism, vascular structure, function and insulin signalling. Additionally, numerous epidemiological evidence suggests that diabetes is associated with cognitive impairment [4]. This association promotes the resistance of insulin, signalling of insulin growth factor (IGF), oxidative stress, neurofibrillary tangle formation, amyloid-beta Aβ formation and regulation of acetylcholine esterase activity [2]. The recent concept suggests that early development of cognitive dysfunction is caused by the deficiency of glucose utilisation by the brain [2, 4, 5]. In addition, a human post-mortem study disclosed IGF and insulin resistance/impairments in signal transduction mechanism [6]. Brain insulin resistance is generally established due to low insulin or/and IGF receptors levels, while deficiencies of insulin and IGF are associated with expression changes in the brain [7]. Researchers termed Alzheimer’s disease “type-3-diabetes” because of shared mechanisms among type-1-diabetes and type-2-diabetes [8]. Diabetic patients have more prevalence of cognitive decrease and a higher risk of developing different types of dementia [9]. This cognitive decline in diabetic persons mainly affects learning behaviour and memory, psychomotor efficiency, attention, mental speed and flexibility, and executive function [10].

Currently, the main focus of the research is toward the evaluation of insulin resistance and insulin deficiency as potential mediators of neurodegeneration. Primely, diabetes causes oxidative stress, brain insulin resistance, and cognitive impairment [11]. Extensive deficiency in brain insulin and IGF signalling leads to early and progressive disturbance, which could be responsible for the biochemical, molecular and/or histopathological lesions in Alzheimer’s disease [7, 8, 12]. The streptozotocin (STZ) induced model in rodents is generally used for diabetes studies [13]. STZ is a glucosamine-nitrosourea compound that causes β-cells cytotoxicity in pancreatic islets, causing insulin deficiency [14]. Numerous reports documented that STZ is responsible for the increase of Aβ oligomers levels in rodent's brains and diabetes [2, 4, 14]. Evidence suggests that soluble Aβ oligomers induce synaptic dysfunction. Additionally, it is also reported in the literature that STZ also increases the levels of β-secretase in the brains of rodents without any significant changes in mRNA levels [2, 14]. These reports evidence the effects of insulin deficiency leading to Aβ abnormalities in the brain caused by STZ.

Natural products have long been and still are the source of treatment and prevention of different disorders, including cancer. Increasing emphasis has been focused on the research on traditional medicine, including many herbs and plants. This requires new approaches to overcome diseases, and thus natural products could be efficacious sources for the prevention and treatment of these diseases [15–20]. Lettuce (*Lactuca sativa*) is a leafy vegetable belonging to the Asteraceae family known for its medicinal properties [21]. Its value in the human diet has been documented for various health-promoting and nutritive constituents, such as phytochemicals, calcium, vitamin A, C, E and iron, which are known to prevent multiple diseases [22]. The seed oil of *L. sativa* has been reported for hypnotic, sedative, analgesic, and anticonvulsant properties along with anti-nociceptive and anti-inflammatory activities [23]. Adesso et al. [24] reported a detailed study showing the effect of lettuce extracts on the iNOC and COX-2 pathway. This study indicated that *L. sativa* extracts strongly inhibit the expression of COX-2, TNF-α and IL-6, which are the inflammatory cytokines by inhibiting the pro-inflammatory transcription factor, P65 NF-ҡB translocation in J774A.1 macrophage cell line [24].

In a former study conducted by our group, in vitro bioassays showed that rol genes (rol A and rol C) significantly enhanced the capability of lettuce to inhibit dipeptidyl peptidase-4, α-glucosidase and stimulated GLP-1 secretion [25]. The ethanolic extract of *L. sativa* has been reported for its shielding effects against D-galactose-mediated oxidative damage in neurons, inhibition of H_2_O_2_-induced neurotoxicity and protection against ischemia-induced neurotoxicity [26]. A research stated that due to scavenging activity and greater phenolic content, an aqueous extract of the lettuce seeds guards against cell death and Aβ-mediated oxidative stress in hippocampal neurons [27]. Lettuce is also reported to reduce lipofuscin granules and protect against oxidative stress [28]. Extract of lettuce on memory was evaluated through Morris water maze and elevated plus-maze tasks for object recognition in addition to biochemical factors [29]. This study revealed that lettuce has the potential for memory enhancement against scopolamine-induced memory deficiencies.

The chemical analysis studies of lettuce confirmed the presence of lactucin, lactucopicrin, deoxylactucin, 15-oxalyl and 8-sulfate conjugates of the guaianolide and sesquiterpene lactones, along with polyphenols like chlorogenic acid, epicatechin, vanillin, caffeic acid, sinapic acid, rutin hydrate, p-coumaric acid, quercetin-3-rhamnoside and quercitin [25, 26, 28]. The medicinal properties of *L. sativa* are believed to be due to the presence of these components. However, detailed mechanisms of these components are yet to find out. Considering the beneficial effects, the present study was aimed to investigate the impact of hydroponically (HyL) and soil-grown (SoL) lettuce on anti-oxidant defence systems, enzyme inhibition, cytotoxic response, streptozotocin-induced diabetes and aluminium chloride-induced neurotoxicity in rats.

## Methods

### Plant cultivation and extract preparation

The seeds of Grand Rapids (cv) lettuce (*Lactuca sativa*) were bought from Awan Seed Store, Rawalpindi, Pakistan and voucher-128,085 was submitted in the “Herbarium of medicinal Plants of Pakistan Quaid-i-Azam University Islamabad”. Plant cultivation under study was carried out as per guidelines of National Environmental Policy [30]. For organic lettuce, seeds were germinated in a mixture of agricultural soil, and sand (3:1) watered using half-strength Hoagland’s solution at 24 °C maintained at 8/16 night/day photo-period. On the other side, *L. sativa* plants were grown hydroponically using a Hydroponics Film Kit under greenhouse conditions at 24 °C. The aerial part was harvested from hydroponically grown lettuce (HyL) and soil-grown lettuce (SoL) for the experiment. The material was dried under shade, ground to powder, and macerated in analytical grade methanol. After frequent shaking/mixing for 5 days, solvents were filtered using Whatman#1 filter paper. The filtrate was dried at 40 °C under a vacuum hood to obtain the methanolic extracts of HyL and SoL, respectively. These extracts were kept at − 20 °C for further processing.

### In vitro assessment

#### Total phenolic contents (TPC)

TPC were measured with Folin-Ciocalteu reagent as reported previously by Ismail et al. [31]. For the experiment, 4 μl (1 mg/ml) of each extract was added in wells of 96-well plate, and 98 μl of Folin-Ciocalteu reagent (10-fold diluted) was mixed. The reaction mixture was incubated at room temperature for 5 minutes, and 98 μl of sodium carbonate (6%) was added. The resulting mixture was incubated at 25 °C for 90 min, and absorbance was recorded at 725 nm with a BioTek plate reader (Elx 800). Total phenolic contents were expressed as Gallic acid equivalents.

#### Total flavonoid contents (TFC)

The aluminium chloride colourimetric method was performed to estimate TFC as described previously [32]. Four microliter of each sample (1 mg/ml) were independently mixed with 10 μl aluminium chloride (10%), 10 μl of potassium acetate (1 M) and 176 μl dH_2_O in wells of 96-well plate. The resulting mixture was incubated at room temperature for 30 min, and absorbance was recorded at 405 nm with a BioTek plate reader (Elx 800). Total flavonoid contents were expressed as Quercetin equivalents.

#### Total anti-oxidant capacity (TAC)

TAC of the extracts was calculated by the phosphomolybdenum method as reported by Kayani et al. [33]. In reaction mixture 4 μl (1 mg/ml) of each extract was added with 196 μl of reagent solutions (sodium phosphate (28 mM), ammonium molybdate (4 mM) and sulfuric acid (0.6 M)). The reaction mixture was then incubated for 90 min at 95 °C in the water bath, and absorbance was recorded at 630 nm with a BioTek plate reader (Elx 800). The anti-oxidant capacity of extracts was expressed as ascorbic acid equivalent.

#### Total reducing power (TRP)

The TRP of the extracts was investigated by Sajid et al. [34]. The assay was performed with 20 μl (1 mg/ml) of extract added with 490 μl of phosphate buffer (0.2 M) and 490 μl of potassium ferricyanide (1%). The mixture was then incubated for 20 min at 50 °C. After that, 500 μl of trichloroacetic acid (10%) was mixed, and the mixture was centrifuged for 10 min at 3000 rpm. Five hundred microliter from the upper layer was taken and mixed with 100 μl of ferric cyanide (0.1%). Then absorbance was recorded at 630 nm with a BioTek plate reader (Elx 800). The reducing power of extracts was expressed as ascorbic acid equivalent.

### Anti-oxidant assays

#### DPPH assay

The anti-oxidant potential of extracts to scavenge the DPPH free radicals was investigated by the method described by Ismail et al. [35]. One hundred ninety-five microliter freshly prepared DPPH (0.1 mM) solution was mixed with 5 μl of each extract (1, 0.5, 0.25 mg/ml) in 96-well plate respectively. The plates were then incubated in the dark at 37 °C for 30 min, and absorbance (Abs) was recorded at 515 nm with BioTek plate reader (Elx 800), and percentage inhibition was calculated. The experiment was performed in triplicate, and ascorbic acid was a positive control.

#### Lipid peroxidation assay

The previously reported method was used to measure lipid peroxidation activity [36] with modifications. Liposomes were prepared through ultrasonic irradiation from egg lecithin by adding 490 μl of FeCl_3_ (400 mM) and 490 μl of ascorbic acid (200 mM). Then 20 μl of sample (1, 0.5, 0.25 mg/ml) was added. The mixtures were incubated for 60 min at 37 °C, and 0.25 M HCl was added to inhibit the reaction. These mixtures were then placed in a boiling water bath for 15 min, cooled and centrifuged. Two hundred microliter of the resulting solution was taken, absorbance was recorded at 532 nm with BioTek plate reader (Elx 800), and percentage inhibition was calculated.

#### DNA damage assay

According to the method reported earlier, HyL and SoL extracts were tested for its DNA protection property [37]. The reaction was performed in PCR tubes consisting 3 μl plasmid DNA (0.5 μg/ml/3 μl), 5 μl of stock solution of each extract at three concentrations (1, 0.5, 0.25 mg/ml), 3 μl of FeSO_4_ (2 mM) and 4 μl of H_2_O_2_ (30%). Positive control (P) was prepared by adding 3 μl plasmid and 12 μl of 50 mM phosphate buffer (pH 7.4) while the negative control (N) was prepared by adding 3 μl plasmid DNA, 4 μl H_2_O_2,_ 3 μl FeSO_4_ and 5 μl of the buffer. To estimate the pro-oxidant effect of the extract on DNA, a control (X) was prepared by adding 3 μl of plasmid DNA, 5 μl of stock solution of extract and 7 μl of the buffer. The mixtures were incubated in the dark for 1 h at 37 °C, and the mixture was run on 0.9% agarose gel to check the DNA protective/damaging effect. The gels were visualised under the Gel-Doc system (BioRad), and percentage inhibition was calculated.

#### Antidiabetic assays

The in vitro antidiabetic assessment was carried out using α-glucosidase and α-amylase inhibition assays on lettuce extracts in a 96-well microtiter plate by the previously reported method [38], for experiment two 96-well plates were prepared independently having 10 μl (0.2 U) of respective enzyme solution, 10 μl substrate (*p*-nitrophenyl-α-d-glucopyranoside for α-glucosidase and starch for α-amylase) 10 μl of each extract with the final concentration of 1, 0.5, 0.25 mg/ml and 70 μl of the buffer. Solutions were mixed well, and plates were incubated at 37 °C (α-glucosidase) and 50 °C (α-amylase) for 30 min. After incubation, 100 μl of sodium bicarbonate (0.5 mM) and HCl (0.2 M) solution were added in α-glucosidase and α-amylase plates, respectively, to stop the reaction. Additionally, in α-amylase plate, 100 μl of iodine reagent (5 mM KI and 5 mM I_2_) was added. Experiments were performed in triplicates using acarbose as the positive control. Absorbance in α-glucosidase and α-amylase plates were measured at 405 nm and 540 nm, respectively, with a microtiter plate reader (BioTek, Elx 800) and percentage inhibition was calculated.

#### Cholinesterase assays

Acetylcholinesterase enzyme plays a vital role in nerve transmissions, while butyrylcholinesterase is involved in detoxification mechanisms. The cholinesterase inhibition assays were performed using Ellman’s method with modification [39–41]. The experiment consisted of 25 μl of respective enzyme (0.2 U), 25 μl (15 mM) substrate (acetylthiocholine iodide for acetylcholinesterase and butryllthiochloline iodide for butyrylcholinesterase), 50 μl (0.1 M) sodium phosphate buffer (pH 8.0), 125 μl (3 mM) DTNB and 25 μl of extract with final concentrations of 1, 0.5, 0.25 mg/ml in wells of 96 well plate respectively. The plates were then incubated at 37 °C for 30 min. Galantamine hydrobromide was used as the positive control, and change in absorbance was recorded at 405 nm with a microplate reader (BioTek, Elx 800), and percentage inhibition was calculated.

#### MTT cytotoxicity assay

MTT (3-(4,5-dimethylthiazol-2-yl)-2,5-diphenyltetrazolium bromide) test was performed to study the antiproliferative activity of lettuce by the previously reported method [42] using MCF7 and HePG2 cell lines. For cytotoxicity assay, 7 × 10^4^ cells/well for the MCF7 line and 3.4 × 10^4^ cells/well for HepG2 and HeLa lines were seeded in a 12-well plate, respectively. MTT assay calculated the viability of the cells by taking absorbance at 570 nm after 48 h in a UV2310 spectrophotometer (Dinko, Spain).

### In vivo assessment

#### Animal grouping and induction of disease

Healthy 6–7 weeks old Sprague Dawley male rats (180-200 g) were obtained from NIH Islamabad, maintained under hygienic conditions, and provided a standard diet at the primate facility. All experiments and methods were approved by the Institutional Ethics Committee of the University of Gujrat, Pakistan, with reference#321 (dated: 18-12-2020), while the Institutional Biosafety Committee approved the animal model of the University of Gujrat, Pakistan with reference#320 (dated: 18-12-2020). During the experiment, minimal animal suffering was ensured as per National Institute of Health (NIH) and ARRIVE guidelines for the care and use of laboratory animals [43]. Total 70 rats were carefully divided into control and treatment groups, containing seven rats. To induce diabetes, 0.2 ml of intraperitoneal injection of 30 mg/kg streptozotocin (STZ) was injected in each rat for three consecutive days [44]. Blood glucose measurement was performed by taking a small drop of blood from the tail of each rat daily with the help of Vita™ test meter (Lifescan). Rat showing glucose level > 300 mg/dl were included in the study. After diabetes induction, aluminium chloride (AlCl3) was administered daily for 3 weeks [44] to induce Alzheimer’s disease symptoms. All groups were administered orally with drugs or samples for 3 weeks representing group-I as healthy control (received saline), group-II served as diabetic control (received STZ), group-III served as a diabetic positive control (received STZ + Glibenclamide), group-IV served as Alzheimer positive control (received STZ + AlCl3 + Rivastigmine) and groups V-X served as experimental groups for HyL and SoL which received STZ + AlCl3 with extract concentration of 200, 100 and 50 mg/Kg of rat body weight respectively.

#### Acute oral toxicity

As per OECD guidelines #425 [45], acute toxicity studies were carried out. For this purpose, the rats were subjected to a single dose (400 mg/kg) orally with extracts of HyL and SoL (seven rats in each) dissolved in saline along with the control group (seven rats) receiving simple saline solution (10 ml/kg). In behavioural parameters, rats feeding pattern, an inspection of skin and body for developing any lesion, skin shredding, housing habits and sleeping pattern were monitored. Any behavioural changes, toxicity or mortality, were observed for up to 1 week.

#### Elevated plus maze test (EPM)

EPM test was carried out to determine the anxiety-like behaviour of HyL, and SoL extracts as reported earlier [44]. Briefly, each rat was placed in a central position facing the open arm and the movement in the maze model was recorded for 5 min with the help of fixed camera. The time spent in close and open spaces was calculated by computer software ANY-maze. A pass was counted when 85% of the rat’s body was on a particular arm. In the end, 70% ethanol and white cloth were used to clean the maze to get it ready for the next rat. After completing the experiment, rats were returned to their cages, and no other experiment was performed on the same day.

#### Morris water maze test (MWM)

MWM test was performed to evaluate the learning and memory behaviour of HyL and SoL extracts as reported previously [44]. The test consisted of one visible and one hidden day test and a single probe trial at the end of hidden tests. The platform was 1 cm above the water surface during visible tests, and rats were placed in all quadrants until they reached the platform. In hidden tests, the platform was placed underwater with added dye to make it invisible. The platform was placed randomly in different quadrants, and rats were released to search and reach the platform. The time consumed in a particular quadrant was recorded for 60 s with the help of a camera. Three trials were performed for each visible and hidden trial with an interval of 1 h, and videos were analysed using ANY-maze software.

#### Serum and tissue preparation

After 21 days, the rats were anaesthetised with isoflurane. The rats were placed separately in an induction compartment, and anaesthesia was induced with 5% isoflurane in oxygen until loss of reflexes. Then the rats were dissected, and blood was collected in BD Vacutainer® tubes. To separate the serum, blood was centrifuged for 10 min at 3500 rpm. The brain, pancreas, liver and kidney tissues were isolated and homogenised separately in ice-cold 50 mM tris buffer at pH -7.4 to estimate organ biomarkers and anti-oxidant enzymes. The brain tissues were stored in saline at − 20 °C for neurotransmitters quantification by HPLC.

#### Quantification of serum amylase and insulin level

Serum amylase was estimated as per the manufacture’s guidelines using standard Cobas® kits (Roche, USA). In contrast, serum insulin levels were determined with the help of the AmgenixMicro ELISA kit (USA) as per the supplier’s guidelines.

#### Determination of serum biochemical markers of liver and kidney

Biochemical markers of the liver (aspartate aminotransferase (AST), alanine aminotransferase (ALT) and total bilirubin) and kidney (blood urea nitrogen (BUN) and creatinine) of respective organs were estimated in the serum samples according to the supplier’s guidelines using Cobas® kits (Roche, USA).

#### Quantification of anti-oxidant enzymes

The Bradford method was used to quantify total protein in homogenised tissue samples by using the Folin Ciocalteu reagent as reported earlier [46]. The homogenates of the brain, liver, pancreas and kidney were analysed for the GSH (reduced glutathione), CAT (catalase), SOD (superoxide dismutase), GPOD (glutathione peroxidase) and TBARS (thiobarbituric acid reactive substances) assays spectrophotometrically for the quantification of enzyme contents. Briefly, TBARS activity was calculated by the previously reported method by Ohkawa et al., [47]. In this method tissue extracts were appropriately mixed with phosphate buffer, trichloroacetic acid (10%) and TBA as working agent consisting of acetic acid (5%) and 20% NaOH. The TBARS activity was measured as mM/min/mg tissue. Aebi’s method was used to estimate CAT activity in which homogenate was mixed with phosphate buffer and hydrogen peroxide, and results were presented as U/min [48]. For GSH activity, sulfosalicylic acid, tissue homogenate phosphate buffer and DNTB were mixed, and activity was calculated as mM/g protein as reported earlier [49]. Bannister method was used to measure SOD activity. Tissue extracts were mixed with phenazine methosulphate and phosphate buffer for the test. Then NADH and glial acetic acid were added. After incubation reading was taken by spectrophotometer, the results were expressed as U/mg protein [50]. Finally, GPOD activity was quantified using 4-methyl catechol (4MC) as substrate. The change in the absorption due to oxidation of 4MC by H_2_O_2_ was measured spectrophotometrically at 420 nm, and results were quantified as U/min [51].

#### High-performance liquid chromatography (HPLC)

Quantification of brain neurotransmitters (serotonin and dopamine) was performed by HPLC as reported previously [44]. Briefly, homogenised tissue samples were centrifuged at 13000 rpm for 12 min at 4 °C following filtration through a 0.2 μm syringe filter. For analysis, 20 μl of the filtrate was injected into HPLC (Agilent: 1200 series). Dopamine and serotonin levels were quantified using a C18 (5 μm × 250 mm × 4.6 mm) column equipped with a UV detector at 270 nm. For clear separation, an isocratic mode mobile phase of 50 mM potassium phosphate buffer (pH -3.5) and methanol 97/3 (v/v) was used at a flow rate of 1.5 ml/min with a total run time 25 min. Final quantification was performed by calculating the peak area of each tissue sample with the linear regression equation obtained from standard curves of dopamine and serotonin.

#### Statistical analysis

The data were analysed by ANOVA following multiple comparison Post-Hoc tests using GraphPad Prism-8. Results are presented in mean ± standard deviation (S.D.) with statistical significance *p* < 0.05.

## Results and discussion

### In vitro assessment

#### Phytochemical and anti-oxidant contents

**Fig. 1:**
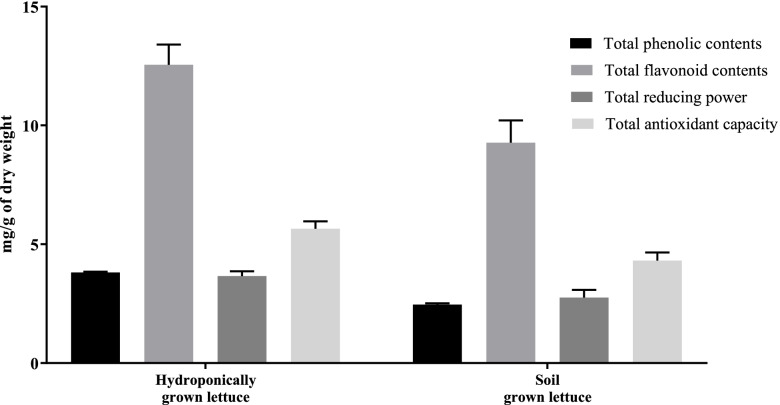
Total phytochemical and anti-oxidant contents. Values expressed as means (*n* = 3) ± SD

TPC and TFC are presented in Fig. 1 as mg of Gallic acid and Quercetin equivalents per 1 g of plant dry weight, respectively. Results demonstrated that in both cases, HyL extract contained the highest level of TPC and TFC as compared with SoL. Phytochemicals including phenolic and flavonoid are vital anti-oxidants that reveal free radicals scavenging activity [52]. TAC was investigated spectrophotometrically through phosphomolybdenum assay, which is centred on the reduction of Mo (IV) to Mo (V) following the formation of green Mo/phosphate (V) compounds at acidic pH. TAC is a quantitative method, and its anti-oxidant efficiency is represented as the number of ascorbic acid equivalents per 1 g of plant dry weight [53]. Results indicated that HyL extract has higher TAC than SoL (Fig. 1). TRP is also an indicator of potential anti-oxidant activity. In TRP, anti-oxidants in the extracts would reduce iron through electron donation [54]. The results showed that both HyL and SoL had good reducing power capability (Fig. 1), expressed as the number of ascorbic acid equivalents per 1 g of dry plant weight. The activity pattern of TRP was similar to TAC. Flavonoids and phenols are well documented for their capability to donate electrons. Moreover, it has been previously reported that the Mo reduction capacity of plans might be depending on their phytochemicals [55]. To confirm this hypothesis, we determined the correlation between HyL and SoL extracts with respective TPC, TFC, TAC and TRP contents by using GraphPad Prism 8, which showed a positive relationship with an R^2^ value of 0.9874 (*p* < 0.01).

#### Anti-oxidant assays

**Fig. 2:**
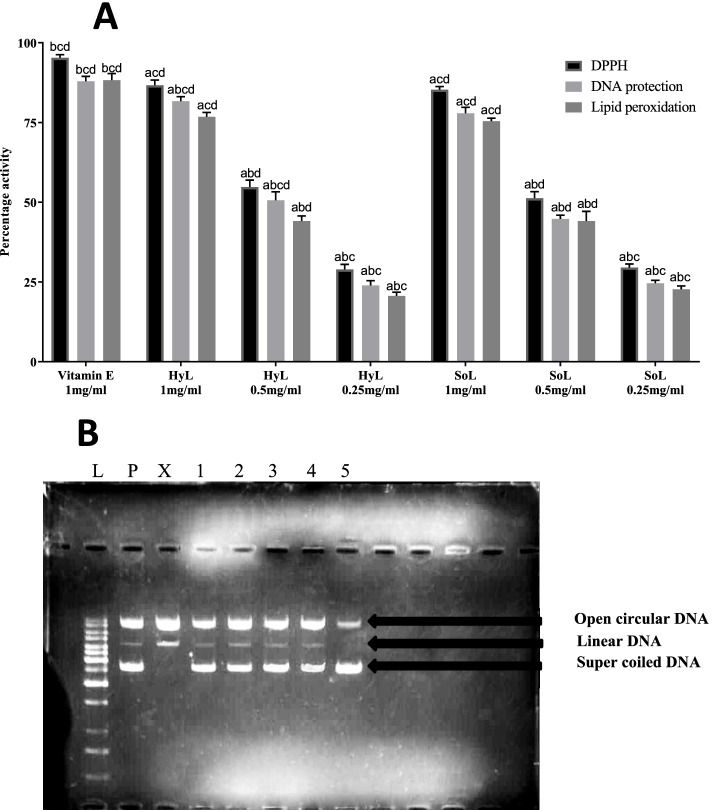
Antioxidant activities (**A**) and DNA protection activity at 1mg/ml (**B**) of hydroponically (HyL) and soil grown (SoL) lettuce. Values expressed as means (*n* = 3) ± SD with *p < 0.05 in comparison with positive control representing “a”, SoL 1 mg/ml representing “b”, SoL 0.5 mg/ml representing “c” and SoL 0.25 mg/ml representing “d” with all respective concentrations of HyL and controls. [Lane# L = DNA ladder (1Kb), P = pBR322 plasmid, X = pBR322 plasmid treated with FeSO4 and H2O2 (control), 1 = pBR322 plasmid + 1 mg/ml of hydroponically grown lettuce; control for the pro-oxidant effect of the extract on DNA, 2 = plasmid + 1 mg/ml of hydroponically grown lettuce + FeSO4 + H2O2, 3 = pBR322 plasmid + 1 mg/ml of soil grown lettuce; control for the pro-oxidant effect on DNA, 4 = plasmid + 1 mg/ml of soil grown lettuce + FeSO4 + H2O2, 5 = plasmid + 1 mg/ml of Vitamin E + FeSO4 + H2O2]

DPPH method is a simple and effective way for the comparative assessment of anti-oxidant activity in different plant extracts. Upon reduction, DPPH changes its colour from violet to yellow, which is the basis of activity detection [11, 42]. Vitamin E was a positive control, which showed 95.3% scavenging activity. HyL and SoL extracts showed 86.7 85.3% DPPH radical scavenging capacity respectively (Fig. 2a). The results show that these extracts are rich in active radical scavengers, like phenolic, flavonoids, and derivatives. So it can be proposed that the DPPH scavenging potential of the lettuce might involve the active role of phytochemicals [56].

In the lipid peroxidation test, thiobarbituric acid interacts with malondialdehyde, a side product of peroxidation producing red chromogen. This red chromogen is measured spectrophotometrically to determine the anti-oxidant potential [57]. The result showed significant anti-oxidant activity for both samples, as shown in Fig. 2. The results presented 81.7, 77.8 and 87.9% activity respectively for HyL, SoL and Vitamin E. Multiple sources like organic-hydro peroxides, redox-cycling and iron-containing compounds may trigger the lipid peroxidation by producing free radicals [58]. The flavonoids reported in this plant may play a positive role as an anti-oxidant by capturing these free radicals.

DNA damage assay is based on the potential of sample protection against the •OH attack generated from the Fenton reaction. This Fenton reaction breaks supercoiled DNA into open circular and/or linear forms [31, 32]. The anti-oxidant and pro-oxidant effects of extracts were evaluated by inspecting the intensity of gel bands formed on 1% agarose. Figure 2b represents the DNA protection effect of HyL and SoL at 1 mg/ml. The presence of a relaxed form of DNA after HyL, SoL and Vitamin E represents 76.8, 75.4 and 88.3% DNA protection activity, respectively (Fig. 1a). Results showed low intensity in nicked DNA formation and high intensity in native DNA form, presenting a significant protection phenomenon. The protective effect of the extract on DNA can inhibit ROS generation by forming a complex with cations participating in •OH formation [59]. The comparison for results between HyL and SoL showed no significant difference at concentration level. However, results are statistically significant with *p* < 0.05 compared with positive control.

#### Enzyme inhibition assays

**Fig. 3:**
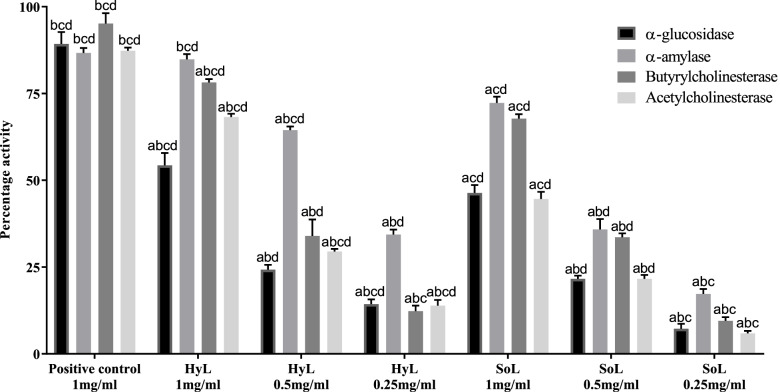
Enzyme inhibition activities of hydroponically (HyL) and soil-grown (SoL) lettuce. Values expressed as means (*n* = 3) ± SD with *p < 0.05 in comparison with positive control representing “a”, SoL 1 mg/ml representing “b”, SoL 0.5 mg/ml representing “c” and SoL 0.25 mg/ml representing “d” with all respective concentrations of HyL and controls. In glucosidase and amylase acarbose, butyrylcholinesterase and acetylcholinesterase galantamine hydrobromide served as the positive control

HyL and SoL extracts were investigated for their potential to inhibit the α-glucosidase enzyme. Acarbose served as a positive control (89.2%), and results are presented in Fig. 3. HyL and SoL exhibited moderate activity with inhibition of 54.3 and 46.3% respectively. Inhibitors of α-glucosidase can prevent the release of free glucose from carbohydrates, which reduces blood glucose levels and causes suppression of hyperglycemia condition [60]. Recently, multiple efforts have been carried out to identify strong and effective inhibitors of α-glucosidase from natural resources [61]. Many flavonoids, alkaloids, anthocyanins, terpenoids, glycosides and phenolic compounds have been identified as α-glucosidase inhibitors [61]. So, the presence of high phytochemical contents in lettuce might be responsible for potential α-glucosidase inhibition.

α-amylase is involved in starch digestion to provide free glucose to the system. The inhibition mechanism is a strategy by preventing starch digestion via blockage of α-amylase. This strategy is used to treat periodontal diseases and diabetes [62]. Plants are the prime source of natural inhibitors against α-amylase. For this purpose, the α-amylase inhibition potential of lettuce extracts was screened spectrophotometrically. The assay was performed in triplicate, and the result in the form of inhibition (%) are presented in Fig. 3. Acarbose served as a positive control with 86.7% inhibition. Plant-based compounds like glycosides, alkaloids, galactomannan gum, polysaccharides, peptidoglycans, hypo glycans, guanidine, glycopeptides, steroids and terpenoids are also reported for anti-hyperglycemic activity [63]. A group of researchers [64] explored the interactions between human α-amylase and flavonoids to understand the fundamental molecular prerequisite for the enzyme inhibition process. They found that the strength of inhibition is associated with the number of OH groups present on the flavonoid skeleton. This finding suggests the involvement of flavonoids in the enzyme inhibition mechanism.

The lack of acetylcholine is the principal characteristic of Alzheimer’s disease, and it contributes to significant symptoms like cognition and decline in memory [65]. In this study, we tested HyL and SoL extracts for inhibition against acetylcholine enzyme by Ellman’s method [39]. The main advantages of Ellman’s method are rapid processing, simplicity and relatively low cost [66]. Percentage inhibitions of 68.2, 44.6 and 87.3% for HyL, SoL and galantamine hydrobromide, respectively, are presented in Fig. 3. It is well known that neurodegenerative disorders are primarily associated with degeneration of the cholinergic system [65]. This can be supported by either using muscarinic agonists or by acetylcholinesterase inhibitors. Enzyme inhibitors are generally responsible for increasing the concentration of acetylcholine [67]. The enzyme inhibition potential of lettuce suggests that this plant has some active ingredients which block the acetylcholinesterase, which requires further studies for identification.

Butyrylcholinesterase is a unique enzyme involved in neural brain function [68]. Like acetylcholinesterase, the extracts of HyL and SoL were tested for butyrylcholinesterase inhibition, a serine-based hydrolase that catalyses the hydrolysis of choline ester's hydrolysis, including acetylcholine. The experiment was performed in triplicates and controls, and the results are summarised in Fig. 3. HyL extract showed good (78.2%) while SoL exhibited moderate (67.7%) activity. Galantamine hydrobromide served as a positive control (95.2%). Our results summarise the biochemical effect of HyL and SoL on butyrylcholinesterase which may have roles in cholinergic detoxification. HyL showed slightly higher activity than SoL at different concentrations in comparing concentrations. However, results are statistically significant with *p* < 0.05 at all concentrations for HyL and SoL.

#### MTT cytotoxicity assay

**Fig. 4:**
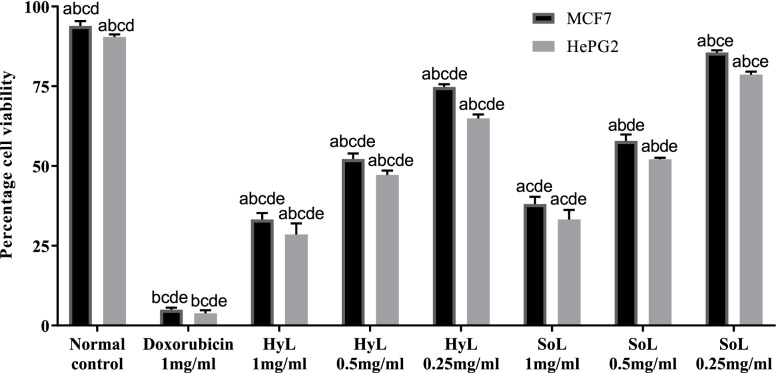
Cytotoxic potential of hydroponically (HyL) and soil-grown (SoL) lettuce. Values expressed as means (*n* = 3) ± SD with *p < 0.05 in comparison with doxorubicin representing “a”, SoL 1 mg/ml representing “b”, SoL 0.5 mg/ml representing “c”, SoL 0.25 mg/ml representing “d” and normal control representing “e” with all respective concentrations of HyL and controls

Cytotoxic effects of HyL and SoL against MCF7 and HePG2 cancer cell lines were measured, and results in the form of percentages are presented in Fig. 4. Doxorubicin was used as the positive control, which showed maximum activity (3.8 and 5.0%) for both cell lines (HePG2 and MCF7). The highest activity was calculated for HyL extract against HeLa (28.6% cell viability) followed by SoL extract (33.2% cell viability). In the case of MCF7, the HyL extract showed 33.3% cell viability, while SoL extract exhibited 38.0% cell viability. The elevated level of free radicals is reported in cancer patients, which may exaggerate the disease state, and it is expected that anti-oxidants have a reverse correlation with cancer [69]. Previously, ethanol extracts of different plants were reported beneficial against HeLa cell line, P388 leukaemia and moult-4-human leukaemia, respectively [70, 71]. Similarly, organic and aqueous fractions of plants are also known to have a protective effect against hydrogen peroxide-induced cytotoxicity in H9c2 cells [72]. Moreover, flavonoids are recently investigated to delay, prevent/help in curing cancer, probably by blocking vulnerable mutations in DNA that may occur in tumour suppressors or oncogenes genes [69]. Our results also showed a strong positive correlation between the MTT assay and flavonoids. Based on our findings, it may be proposed that flavonoids have some mechanistic role in cancer suppression which needs to be explored.

### In vivo assessment

#### Acute oral toxicity

No mortality and behavioural changes were recorded in the experimental rats for 1 week. Overall, rats feeding patterns remained unchanged, their skin and body were inspected for any lesion or skin shedding, but no sign of abnormality was observed. After completing the acute oral toxicity assay, serum assays like ALT, AST, BUN, creatinine, and total bilirubin were performed by taking a small amount of blood from the tail vail. The levels of these markers were found normal. The present study acknowledges the limitations for the fact that the histological presentations of the vital organs and whole blood profiles were not performed and that they should be essentially investigated in the future for validation of the acute oral toxicity profiles.

#### Elevated plus maze test (EPM)

EPM assay was performed to investigate the anxiety-like response of rats after the induction of Alzheimer. Data were recorded in the form of distance travelled in open and closed arm zone, and results are shown in Table 1. Alzheimer’s control group significantly reduced distance travelled in open arms (33.3 cm) compared to the normal control group (93.7 cm). In comparison, Rivastigmine (positive control) group exhibited a substantial increase (83.4 cm) in the travelled distance. Alzheimer control group showed an increase in total travelled distance (165.0 cm) in closed compared with the normal control group (103.0 cm), while Rivastigmine (positive control) group exhibited a significant decrease (109.6 cm). HyL and SoL treated groups showed prominent responses with total distance travelled in the open arm (63.7 cm and 42.1 cm) and closed arm (87.6 cm and 129.7 cm), respectively (Table 1). Overall, results represent the anti-anxiety behaviour of the HyL and SoL extracts. EPM test detects an anxiolytic potential of different classes of therapeutic drugs without any sophisticated conditioning requirements. This test is based on the inherent conflict affected by the anxiety level. Anxiety and depression can originate due to the oscillation of the role of neurotransmitters, mainly serotonin, noradrenalin and dopamine [20, 73, 74]. MAO inhibitors enhance endogenous amines like serotonin and catecholamine [75], but they are not free from side effects. However, plants like *Plumeria rubra* and *Trichilia catigua* are reported to increase the exploration rate of open arms in EPM being used in folk medicine to diminish anxiety [75–77]. Similarly, our results indicated that the total time spent and the total number of entries in the enclosed arms are prominently reduced compared to the control group, representing an anxiolytic-like effect.

#### Morris water maze test (MWM)

MWM test was performed as a tool to investigate learning/memory responses after the induction of the Alzheimer and results are summarised in Table 1. Results showed a significant reduction in total time spent in the platform quadrant in the Alzheimer control group (3.1 s). The treatment of HyL and SoL significantly increased (10.2 s and 7.7 s respectively) the time in the platform quadrant. The results of Alzheimer’s control signifies that the rats were not adequately oriented in their directions, while in normal control and Rivastigmine (positive control), they were well oriented. Additionally, we investigated the latency of first entry in the platform quadrant, which exhibited significant elevation in time for the Alzheimer control group (30.3 s) compared to the normal control group (8.1 s). However, treatment of HyL (13.2 s) and SoL (22.0 s) exhibited a significant decline in latency time (first entry) in the platform zone, as presented in Table 1. The MWM test is generally reported to evaluate long-term spatial learning capability and reference memory [78]. On the other hand, the escape latency phenomenon is used as a procurement test reflecting spatial learning behaviour. This means that rats need to learn the exact position of the platform hidden in the maze and develop suitable tactics to reach it [79]. Additionally, crossing numbers in virtual-platform is a crucial indicator to assess the reference memory in the absence of a platform. As reported in previous studies [79, 80], both HyL and SoL significantly increased the escape latency in the acquisition phase. They decreased the numbers of crossing virtual-platform representing the memory-enhancing activity.

**Table 1:** Elevated Plus Maze and Morris Water Maze test of hydroponically (HyL) and soil-grown (SoL) lettuce

Sr. No.	Test group	Elevated Plus Maze		Morris Water Maze	
		Closed arm zone (cm)	Open arm zone (cm)	Total time in the platform quadrant (sec)	The first entry in the platform quadrant (sec)
1	Normal control	103.1 ± 2.1^abcde^	93.7 ± 3.9^abcde^	12.7 ± 0.9^bcde^	8.1 ± 1.0^bcde^
2	Alzheimer control	165.0 ± 4.3^abcf^	33.3 ± 4.6^abdf^	3.1 ± 0.5^abf^	30.3 ± 2.2^abcf^
3	Rivastigmine 10mg/kg	109.6 ± 4.2^bcdef^	83.4 ± 3.1^bcdef^	11.6 ± 1.3^bcde^	10.8 ± 1.0^bcde^
4	HyL 200mg/kg	87.6 ± 4.5^abcdef^	63.7 ± 3.9^abcdef^	10.2 ± 1.2^cde^	13.2 ± 1.1^bcde^
5	HyL 100mg/kg	103.0 ± 1.8^abcde^	53.1 ± 1.6^abcdef^	8.4 ± 0.1^def^	19.6 ± 0.2^abcde^
6	HyL 50mg/kg	126.4 ± 1.6^acdef^	43.1 ± 1.6^acdef^	5.3 ± 0.1^af^	22.7 ± 0.2^ade^
7	SoL 200mg/kg	129.7 ± 4.8^acdef^	42.1 ± 4.6^acdef^	7.7 ± 0.6^adef^	22.0 ± 2.3^ade^
8	SoL 100mg/kg	156.1 ± 1.7^abdef^	34.1 ± 1.3^abdf^	6.1 ± 0.1^af^	25.3 ± 0.2^ade^
9	SoL 50mg/kg	167.0 ± 3.2^abcf^	24.0 ± 1.5^abcef^	3.1 ± 0.2^abf^	29.5 ± 0.2^abc^

Values expressed as means (*n* = 7) ± SD with *p < 0.05 in comparison with Rivastigmine representing “a”, SoL 200 mg/kg representing “b”, SoL 100 mg/kg representing “c”, SoL 50 mg/kg representing “d”, Alzheimer’s control representing “e” and normal control representing “f” with all respective concentrations of HyL and controls

#### Determination of plasma glucose levels

STZ was used to induce diabetes mellitus, which is known for causing the cytotoxic effect on pancreatic beta cells. Consequently, STZ disturbs the release of endogenous insulin resulting in elevation of blood glucose level [81]. The impact of HyL and SoL extracts on glucose level in STZ-induced rats was recorded with the help of Lifescan digital glucometer (Vita™), and the results are summarised in Table 2. The diabetic group exhibited a glucose range of 405-528 mg/dl while the glucose level of Glibenclamide (positive control) remained in the range of above 95-105 mg/dl during the 21 days experiment (Table 2). HyL group showed a significant decrease in glucose level from 415 mg/dl on 0 h to 126 mg/dl on 21st day while in SoL moderately decreased the blood from 395 mg/dl on 0 h to 165 mg/dl on 21st day (Table 2). As an alkylating agent, streptozotocin cause fragmentation of DNA [82]. Damages in DNA activate the natural repair process leading to dephosphorylation of ATP, which activates the xanthine oxidase by providing a substrate, finally resulting in ROS formation. The protective action of HyL and SoL was additionally confirmed by DNA damage assay (Fig. 2b), representing ROS formation prevention through protection from possible DNA damage. Moreover, extract's possible mechanisms of action could be associated with potentiating insulin from beta cells or increasing peripheral glucose uptake [82, 83].

**Table 2:** Effect of hydroponically (HyL) and soil-grown (SoL) lettuce on blood glucose level (mg/dL)

Groups	0 h	3^**rd**^hour	6^**th**^hour	12^**th**^hour	2^**nd**^day	6^**th**^day	10^**th**^day	14^**th**^day	18^**th**^day	21^**st**^day
Normal control	99 ± 2.6^abcde^	97 ± 3.1^abcde^	98 ± 2.4^abcde^	95 ± 4.0^abcde^	93 ± 3.4^abcde^	95 ± 3.0^abcde^	92 ± 2.2^abcde^	98 ± 3.6^abcde^	99 ± 1.3^abcde^	97 ± 4.2^abcde^
Diabetic control	405 ± 4.1^bcdf^	528 ± 2.3^abcdf^	510 ± 4.2^abcdf^	515 ± 3.9^abcdf^	505 ± 3.4^abcdf^	501 ± 2.5^abcdf^	490 ± 3.6^abcdf^	495 ± 4.5^abcdf^	492 ± 2.1^abcdf^	489 ± 3.2^abcdf^
Glibenclamide 10mg/kg	410 ± 1.9^bcdf^	131 ± 1.2^bcdef^	105 ± 4.3^bcdef^	103 ± 2.8^bcdef^	101 ± 3.4^bcdef^	105 ± 3.1^bcdef^	95 ± 2.5^bcdef^	97 ± 2.2^bcdef^	96 ± 2.8^bcdef^	97 ± 1.1^bcdef^
HyL 200mg/kg	415 ± 4.2^bcdef^	250 ± 3.2^abcdef^	187 ± 2.9^abcdef^	162 ± 1.9^abcdef^	145 ± 3.0^abcdef^	138 ± 1.8^abcdef^	135 ± 1.4^abcdef^	132 ± 3.1^abcdef^	131 ± 4.2^abcdef^	126 ± 4.4^abcdef^
HyL 100mg/kg	412 ± 5.1^bcdef^	247 ± 4.2^abdef^	225 ± 3.5^adef^	198 ± 3.6^abcdef^	195 ± 4.8^acdef^	190 ± 5.6^abcdef^	185 ± 6.3^abcdef^	183 ± 5.2^abcdef^	183 ± 4.8^abcdef^	180 ± 3.5^abcdef^
HyL 50mg/kg	405 ± 3.6^bcdef^	255 ± 5.1^abcdef^	235 ± 5.7^abcdef^	210 ± 4.9^adef^	205 ± 4.7^abdef^	206 ± 4.3^abdef^	209 ± 4.8^abcdef^	208 ± 5.9^abdef^	203 ± 6.2^abdef^	206 ± 4.2^abdef^
SoL 200mg/kg	395 ± 3.2^acdef^	310 ± 3.4^abdef^	228 ± 2.5^acdef^	206 ± 2.8^adef^	196 ± 3.4^acdef^	184 ± 3.6^acdef^	176 ± 1.9^acdef^	175 ± 1.3^acdef^	172 ± 3.5^acdef^	165 ± 2.4^acdef^
SoL 100mg/kg	310 ± 4.1^abdef^	242 ± 5.0^abdef^	220 ± 4.6^abdef^	210 ± 2.9^adef^	205 ± 6.1^abdef^	203 ± 4.7^abdef^	202 ± 5.1^abdef^	210 ± 4.5^abdef^	200 ± 4.1^abdef^	201 ± 5.7^abdef^
SoL 50mg/kg	350 ± 5.2^abcef^	260 ± 5.6^abcef^	242 ± 6.1^abcef^	235 ± 3.5^abcef^	228 ± 4.8^abcef^	224 ± 5.2^abcef^	232 ± 5.9^abcef^	226 ± 6.1^abcef^	231 ± 5.3^abcef^	231 ± 6.2^abcef^

Values expressed as means (*n* = 7) ± SD with *p < 0.05 in comparison with Glibenclamide representing “a”, SoL 200 mg/kg representing “b”, SoL 100 mg/kg representing “c”, SoL 50 mg/kg representing “d”, diabetic control representing “e” and normal control representing “f” with all respective concentrations of HyL and controls

#### Quantification of serum amylase and insulin level

**Fig. 5:**
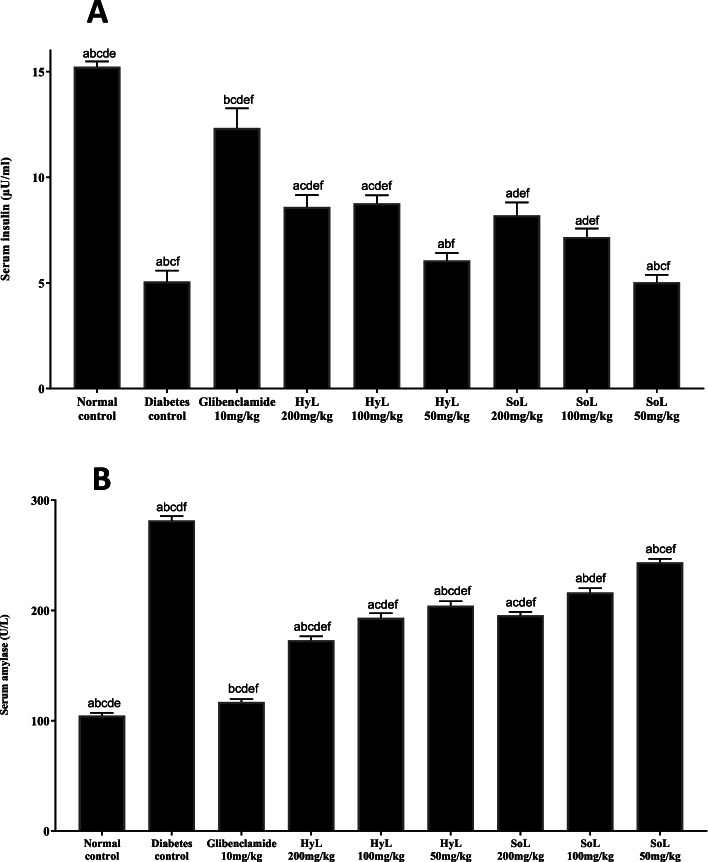
Serum insulin (**A**) and amylase (**B**) levels after treatment with hydroponically (HyL) and soil-grown (SoL) lettuce. Values expressed as means (*n* = 7) ± SD with *p < 0.05 in comparison with Glibenclamide representing “a”, SoL 200 mg/kg representing “b”, SoL 100 mg/kg representing “c”, SoL 50 mg/kg representing “d”, diabetes control representing “e” and normal control representing “f”, with all respective concentrations of HyL and controls

The results of insulin quantification of serum are represented in Fig. 5a. The diabetic control group showed decreased insulin levels compared to normal control, while the diabetic rats treated with Glibenclamide showed enhanced insulin levels. Similarly, the diabetic rats treated with HyL and SoL exhibited significant (*p* < 0.05) but moderately enhanced insulin levels in a concentration-dependent manner. Meanwhile, serum amylase level is shown in Fig. 5b with the following treatment of Glibenclamide, HyL and SoL. The results indicate a significant (*p* < 0.05) increase in the amylase level in the diabetic rats compared with controls. Amylase levels were decreased significantly (*p* < 0.05) in the diabetic rats after the treatment with both HyL and SoL. However, the high dose of HyL and SoL was better than the low.

#### Determination of serum biochemical markers of liver and kidney

The increase of enzyme levels in serum is attributed to liver dysfunction, resulting from the rupture of hepatocytes. As damage to the liver leads to the release of enzyme markers into the bloodstream. This causes either change in cell membrane permeability or necrosis [84]. The levels of blood urea, creatinine and uric acid are routine markers for analysing renal function. These markers can monitor biological/pathologic processes and the roadmap indicators of renal diseases [85]. Serum biomarkers of kidney and liver were evaluated in STZ-induced rats, and results are presented in Table 3. In the case of the liver, the levels of ALT and AST were increased in HyL and SoL extracts while the levels of total protein and total bilirubin levels were decreased compared with the diabatic group (Table 3). A similar pattern was observed in the case of kidney biomarkers in which BUN levels were increased and creatinine levels were decreased after treatment of HyL and SoL extracts as compared with diabatic control group (Table 3). These results are in according with the normal group (Table 3) which represents that the HyL and SoL treatment has positive effects on the liver and kidney biomarkers which help in restoration of all altered levels of biomarkers towards the normal healthy range.

**Table 3:** Effects of hydroponically (HyL) and soil-grown (SoL) lettuce on liver and kidney biomarkers in serum

Groups	Liver			Kidney	
	ALT (U/L)	AST (U/L)	Total bilirubin (mg/dl)	BUN (mg/dl)	Creatinine (mg/dl)
Normal control	20.8 ± 1.0^abcd^	21.9 ± 1.5^abcd^	0.38 ± 0.0^c^	18.8 ± 0.^7abcd^	0.35 ± 0.02^ns^
Diabetic control	55.4 ± 3.8^abcf^	79.1 ± 3.5^abcdf^	1.3 ± 0.06^c^	38.2 ± 2.0^af^	1.5 ± 0.05^ns^
Glibenclamide 10mg/kg	25.8 ± 2.9^bcdf^	28.5 ± 2.2^bcdf^	0.42 ± 0.03^c^	22.4 ± 2.0^bcdf^	0.62 ± 0.02^ns^
HyL 200mg/kg	29.8 ± 2.2^abcdf^	39.5 ± 2.8^acdf^	0.61 ± 0.06^c^	28.1 ± 2.6^abcdf^	0.70 ± 0.07^ns^
HyL 100mg/kg	35.6 ± 3.1^abcdf^	47.3 ± 3.2^abcdf^	0.89 ± 0.03^c^	30.2 ± 3.2^abcdf^	0.95 ± 0.08^ns^
HyL 50mg/kg	45.3 ± 4.4^acdf^	59.3 ± 3.8^abdf^	0.96 ± 0.04^ns^	32.6 ± 2.8^acdf^	1.12 ± 0.09^ns^
SoL 200mg/kg	45.2 ± 1.5^acdf^	42.6 ± 2.8^acdf^	0.85 ± 0.03^c^	35.5 ± 2.3^adf^	1.23 ± 0.09^ns^
SoL 100mg/kg	50.3 ± 4.6^abdf^	60.3 ± 3.7^abdf^	0.99 ± 0.07^ns^	38.2 ± 3.1^af^	1.36 ± 0.26^ns^
SoL 50mg/kg	55.3 ± 5.1^abcf^	72.3 ± 4.9^abcf^	1.32 ± 0.09^c^	40.2 ± 4.2^abf^	1.49 ± 0.34^ns^

Values expressed as means (*n* = 7) ± SD with *p < 0.05 in comparison with Glibenclamide representing “a”, SoL 200 mg/kg representing “b”, SoL 100 mg/kg representing “c”, SoL 50 mg/kg representing “d”, diabetic control representing “e” and normal control representing “f” with all respective concentrations of HyL and controls. “ns” represent non significance

#### Quantification of anti-oxidant enzymes in rat organs

Anti-oxidants play a vital role in the prevention of oxidative damage. In diabetes, the hyperglycaemic condition can deactivate anti-oxidant enzymes by the glycation proteins, consequently producing oxidative stress, leading to lipid peroxidation [86]. The end products of lipid peroxidation are usually determined by measuring TBARS activity, a pioneering lipid peroxidation index [87]. The increased levels of TBARS are reported in the plasma of diabetic patients [88]. The enzymatic and non-enzymatic mechanisms balance the free radical system in the body through the generation of ROS and their neutralisation [89]. Some enzymes like SOD, CAT, GSH and GPOD play an active role in neutralising specific free radicals. Dismutation of superoxide ions is catalysed by the SOD enzyme, which protects the membranes and cellular structures from oxygen-based free radicals [90]. The catalase enzyme carries the canalisation of hydrogen peroxides, which protects tissues against hydroxyl radicals [91]. GSH reacts with lipid peroxidation by-products and is considered a vital component of the anti-oxidant defence system [92], including different glutathione-dependent enzymes. GPOD is also involved in removing lipid peroxidation by-products by catalysing glutathione oxidation and H_2_O_2_ deactivation. The glutathione reductase ensures the reverse glutathione reduction [93].

Together, these enzymes effectively eliminate the toxic free radicals in vivo. Anti-oxidant enzymes in the pancreas, liver, kidney and brain were quantified spectrophotometrically in HyL and SoL treated tissue and results are shown in Table 4. Both samples showed similar behaviour. However, the HyL showed a higher response while the SoL extract showed a moderate response. Overall, the results showed a rise in CAT, SOD, GPOD and GSH in all tissue samples compared with diabetic control. At the same time, the decrease in TBARS activity was recorded in all tissues samples compared with the diabetic group (Table 4). The results exhibited that trend of the values moves toward the normal control group representing the restoration of altered enzymes levels due to the diabetic side effects.

**Table 4:** Effects of hydroponically (HyL) and soil-grown (SoL) lettuce on pancreas, liver, kidney and brain biomarkers in tissue

Organ	Treatment	TBARS (nM/min/ mg tissue)	CAT (U/min)	SOD (U/mg protein)	GPOD (U/min)	GSH (mM/g tissue)
**Pancreas**	Normal control	32.6 ± 1.6^cdef^	5.8 ± 0.04^cdef^	2.9 ± 0.8^ns^	5.6 ± 0.7^cdef^	9.5 ± 0.6^cdef^
	Diabetic control	41.2 ± 2.1^acd^	1.9 ± 0.02^ach^	1.5 ± 0.05^ns^	2.9 ± 0.07^ah^	5.2 ± 0.3^h^
	Glibenclamide 10mg/kg	33.1 ± 1.3^cdef^	5.2 ± 0.05^cdef^	2.7 ± 0.02^ns^	5.0 ± 0.04^cdef^	8.9 ± 0.1^cdef^
	HyL 200mg/kg	36.4 ± 2.5^acdefh^	4.8 ± 0.09^cdef^	2.5 ± 0.07^ns^	4.5 ± 0.8^cde^	8.1 ± 0.5^cdefh^
	HyL 100mg/kg	37.3 ± 3.1^adefh^	3.5 ± 0.05^aeh^	1.9 ± 0.0^ns^	3.8 ± 0.6^cdeh^	7.2 ± 0.4^adefh^
	HyL 50mg/kg	38.4 ± 2.1^aefh^	2.9 ± 0.08^ah^	1.6 ± 0.04 ^ns^	3.2 ± 0.2^aeh^	6.5 ± 0.3^ah^
	SoL 200mg/kg	38.1 ± 1.3^aefh^	2.8 ± 0.04^ah^	1.9 ± 0.06^ns^	2.1 ± 0.6^ah^	6.3 ± 0.8^ah^
	SoL 100mg/kg	39.1 ± 3.3^aefh^	2.2 ± 0.06^ah^	1.5 ± 0.05^ns^	1.9 ± 0.7^ah^	5.5 ± 0.2^ah^
	SoL 50mg/kg	40.9 ± 3.6^acdh^	1.8 ± 0.08^ah^	1.3 ± 0.04^ns^	1.5 ± 0.8^ah^	4.9 ± 0.6^ah^
**Liver**	Normal control	37.3 ± 3.2^cdef^	14.1 ± 1.1^cdef^	5.1 ± 0.03^ns^	5.2 ± 0.03^ns^	10.4 ± 0.5^cdef^
	Diabetic control	55.1 ± 2.9^ac^	10.2 ± 1.2^ah^	3.2 ± 0.01^ns^	4.0 ± 0.03^ns^	5.8 ± 0.3^ah^
	Glibenclamide 10mg/kg	39.8 ± 3.1^cdefh^	13.9 ± 1.0^cdef^	4.9 ± 0.02^ns^	4.9 ± 0.02^ns^	10.1 ± 0.2^cdef^
	HyL 200mg/kg	45.9 ± 3.1^acdefh^	12.4 ± 1.0^cde^	4.1 ± 0.8^ns^	4.5 ± 0.06^ns^	9.6 ± 0.8^cdef^
	HyL 100mg/kg	47.6 ± 4.1^acdefh^	11.3 ± 1.2^ah^	3.6 ± 0.6^ns^	4.1 ± 0.04^ns^	8.2 ± 0.4^e^
	HyL 50mg/kg	50.8 ± 3.6^aefh^	10.5 ± 1.3^ah^	4.5 ± 0.4^ns^	3.8 ± 0.0^ns^	7.3 ± 0.9^ah^
	SoL 200mg/kg	51.2 ± 4.8^aefh^	9.8 ± 1.8^ah^	3.9 ± 0.6^ns^	3.8 ± 0.08^ns^	6.8 ± 0.5^ah^
	SoL 100mg/kg	52.6 ± 5.1^ah^	9.5 ± 1.4^ah^	3.2 ± 0.7^ns^	3.5 ± 0.01^ns^	6.1 ± 0.9^ah^
	SoL 50mg/kg	54.8 ± 3.6^ach^	9.1 ± 1.3^ah^	2.9 ± 0.3^ns^	3.2 ± 0.03^ns^	5.3 ± 0.5^ah^
**Kidney**	Normal control	30.0 ± 3.4^acdef^	13.8 ± 0.7^de^	3.9 ± 0.5^ns^	4.5 ± 0.5^f^	8.0 ± 0.8^cdef^
	Diabetic control	58.2 ± 4.5^acd^	11.0 ± 0.8^ns^	1.4 ± 0.05^ns^	1.2 ± 0.01^ach^	3.3 ± 0.6^ah^
	Glibenclamide 10mg/kg	35.2 ± 3.6^cdefh^	13.3 ± 0.8^de^	3.5 ± 0.02^ns^	4.4 ± 0.01^f^	7.7 ± 0.04^cdef^
	HyL 200mg/kg	40.3 ± 4.6^acdefh^	12.9 ± 0.9^e^	2.6 ± 0.6^ns^	3.9 ± 0.6^ns^	7.1 ± 0.6^cdef^
	HyL 100mg/kg	45.3 ± 4.8^adefh^	12.2 ± 1.3^e^	1.9 ± 0.2^ns^	2.5 ± 0.5^ns^	6.2 ± 0.2^ef^
	HyL 50mg/kg	51.6 ± 5.2^acefh^	11.8 ± 1.2^ns^	4.5 ± 0.1^def^	1.3 ± 0.6^ah^	5.1 ± 0.5^h^
	SoL 200mg/kg	47.9 ± 2.5^adefh^	11.5 ± 1.1^ns^	2.0 ± 0.8^ns^	4.1 ± 0.8^f^	4.2 ± 0.02^ah^
	SoL 100mg/kg	52.7 ± 4.6^acefh^	10.3 ± 1.5^ah^	1.5 ± 0.4^ns^	2.9 ± 0.4^ns^	3.5 ± 0.6^ah^
	SoL 50mg/kg	57.4 ± 4.1^acdh^	9.2 ± 1.2^ah^	1.2 ± 0.2^ns^	1.9 ± 0.1^ns^	2.9 ± 0.4^ah^
**Brain**	Normal control	15.2 ± 2.1^bcdeg^	5.2 ± 0.4^ns^	1.3 ± 0.05^ns^	5.9 ± 0.01^ns^	4.8 ± 0.6^deg^
	Alzheimer control	29.6 ± 3.1^bch^	3.8 ± 0.01^ns^	0.4 ± 0.02^ns^	3.5 ± 0.7^ns^	1.9 ± 0.8^bh^
	Rivastigmine 10mg/kg	18.9 ± 3.4^cdegh^	4.3 ± 0.01^ns^	1.1 ± 0.07^ns^	5.6 ± 0.02^ns^	4.6 ± 0.3^eg^
	HyL 200mg/kg	20.2 ± 3.1^cdegh^	4.0 ± 0.9^ns^	0.9 ± 0.02^ns^	5.0 ± 0.4^ns^	4.2 ± 0.4^ns^
	HyL 100mg/kg	25.6 ± 4.3^bdegh^	3.8 ± 0.6^ns^	0.6 ± 0.01^ns^	4.4 ± 0.1^ns^	3.5 ± 0.6^ns^
	HyL 50mg/kg	28.4 ± 4.6^bch^	4.7 ± 0.8^ns^	0.4 ± 0.02^ns^	3.8 ± 0.5^ns^	2.8 ± 0.2^ns^
	SoL 200mg/kg	25.5 ± 2.9^cbdegh^	5.0 ± 1.3^ns^	0.5 ± 0.01^ns^	4.5 ± 0.03^ns^	2.7 ± 0.8^ns^
	SoL 100mg/kg	28.9 ± 3.9^dbch^	5.3 ± 1.0^ns^	0.3 ± 0.03^ns^	4.0 ± 0.2^ns^	2.2 ± 0.3^h^
	SoL 50mg/kg	30.3 ± 3.4^bch^	5.4 ± 1.2^ns^	0.1 ± 0.01^ns^	3.1 ± 0.3^b^	1.8 ± 0.5^bh^

Values expressed as means (*n* = 7) ± SD with *p < 0.05 in comparison with Glibenclamide representing “a”, Rivastigmine representing “b”, SoL 200 mg/kg representing “c”, SoL 100 mg/kg representing “d”, SoL 50 mg/kg representing “e”, diabetic control representing “f”, Alzheimer’s control representing “g” and normal control representing “h” with all respective concentrations of HyL and controls. “ns” represent non significance

#### Quantification of dopamine and serotonin

**Fig. 6:**
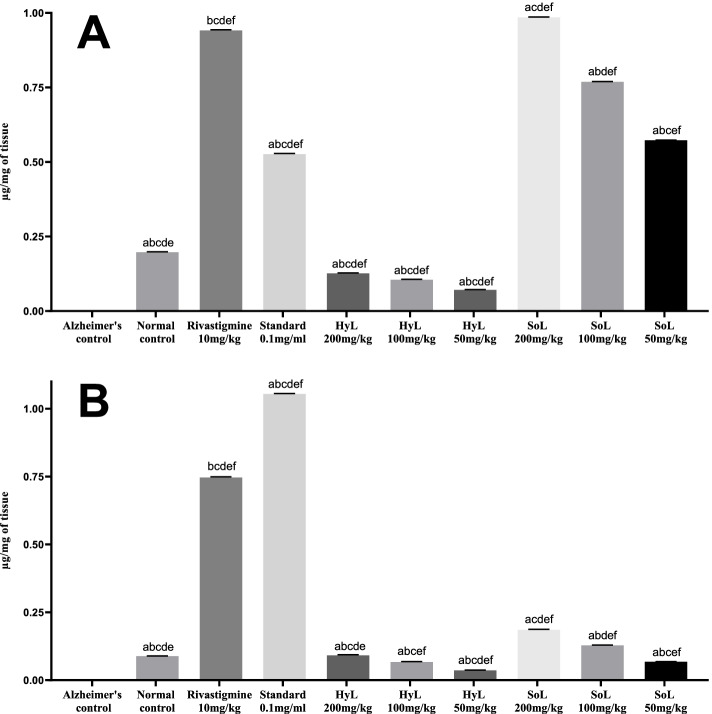
HPLC analysis of (**A**) serotonin and (**B**) dopamine in the brain tissues of rats treated with hydroponically (HyL) and soil-grown (SoL) lettuce. Values expressed as means (*n* = 3) ± SD with *p < 0.05 in comparison with Rivastigmine representing “a”, SoL 200 mg/kg representing “b”, SoL 100 mg/kg representing “c”, SoL 50 mg/kg representing “d” Alzheimer’s control representing “e” and normal control representing “f” with all respective concentrations of HyL and controls

Quantification of neurotransmitters, including dopamine and serotonin, was performed using analytical HPLC-1200 (Agilent). The analysis was performed in triplicate, and results are presented in Fig. 6. First of all, the pure standard of dopamine and serotonin were separately quantified as 1.05419 μg/ml and 0.5266 μg/ml, respectively. The controls exhibited an apparent difference in dopamine and serotonin levels, zero for Alzheimer’s control and highest in Rivastigmine (positive control) compared with the normal control group (Fig. 6a). SoL treatment induced a significant rise with *p* < 0.001 in the level of serotonin (0.9846 μg/mg of tissue), while HyL treatment had a lower effect on the serotonin level (0.1264 μg/mg of tissue). In the case of dopamine, both HyL and SoL showed a moderate but significant increase with *p* < 0.001 in the neurotransmitter level compared with controls (Fig. 6b). Reports claim that this serotonergic system plays a crucial role in interactive learning and memory retention by linking the cholinergic, dopaminergic and glutaminergic systems [94]. Preclinical reports suggest that receptors of these specific neurotransmitters ligands are responsible for increasing neurogenesis, cholinergic neurotransmission, neuronal plasticity and reduction of amyloid burden in the brain [94]. Evidence supports the role of serotonin signalling on Aβ and tau proteins accumulation in the brain [95, 96]. Amyloid precursor protein and tau proteins [97] are known for accumulating clinical symptoms even decades before disease manifestation.

## Conclusion

Overall, *Lactuca sativa* showed antidiabetic, cytoprotective, anti-oxidant and memory enhancement properties. Our study can be used as the baseline for further investigation in finding treatment for diseases like Alzheimer’s, diabetes and cancer. Additionally, we use the HyL approach in this study, which provides a viable alternative to solvent-based whole-plant extraction to produce secondary metabolites.

## Data Availability

All data generated or analysed during this study are included in this manuscript.

## References

[1] Torres N, Avila-Nava A, Medina-Vera I, Tovar AR (2020). Dietary fiber and diabetes. Science and Technology of Fibers in Food Systems.

[2] Kimura N (2016). Diabetes mellitus induces Alzheimer's disease pathology: histopathological evidence from animal models. Int J Mol Sci.

[3] Hashmi WJ, Ismail H, Jafri L, Mirza B (2020). Ethnopharmacological activity of *Hedera nepalensis* K. Koch extracts and lupeol against alloxan-induced type I diabetes. Brazilian. J Pharm Sci.

[4] Kandimalla R, Thirumala V, Reddy PH (2017). Is Alzheimer's disease a type 3 diabetes? A critical appraisal. Biochim Biophys Acta Mol Basis Dis.

[5] Chen X, Zhang M, Ahmed M, Surapaneni KM, Veeraraghavan VP, Arulselvan P (2021). Neuroprotective effects of ononin against the aluminium chloride-induced Alzheimer's disease in rats. Saudi J Biol Sci.

[6] Arnold SE, Arvanitakis Z, Macauley-Rambach SL, Koenig AM, Wang H-Y, Ahima RS (2018). Brain insulin resistance in type 2 diabetes and Alzheimer disease: concepts and conundrums. Nat Rev Neurol.

[7] Nakabeppu Y. Molecular pathophysiology of insulin depletion, mitochondrial dysfunction, and oxidative stress in Alzheimer's disease brain. Diabetes Mellitus. 2019;1128:27–44. 10.1007/978-981-13-3540-2_3 31062324

[8] Nguyen TT, Ta QTH, Nguyen TKO, Nguyen TTD, Van Giau V (2020). Type 3 diabetes and its role implications in Alzheimer's disease. Int J Mol Sci.

[9] Pal K, Mukadam N, Petersen I, Cooper C (2018). Mild cognitive impairment and progression to dementia in people with diabetes, prediabetes and metabolic syndrome: a systematic review and meta-analysis. Soc Psychiatry Psychiatr Epidemiol.

[10] van Duinkerken E, Ryan CM (2020). Diabetes mellitus in the young and the old: effects on cognitive functioning across the life span. Neurobiol Dis.

[11] Sajid M, Khan MR, Ismail H, Latif S, Rahim AA, Mehboob R (2020). Antidiabetic and anti-oxidant potential of *Alnus nitida* leaves in alloxan induced diabetic rats. J Ethnopharmacol.

[12] Oguntibeju OO (2019). Type 2 diabetes mellitus, oxidative stress and inflammation: examining the links. Int J Physiol Pathophysiol Pharmacol.

[13] Wang-Fischer Y, Garyantes T (2018). Improving the reliability and utility of streptozotocin-induced rat diabetic model. J Diabetes Res.

[14] Siedlecka D, Micał W, Krzewicka-Romaniuk E (2020). Streptozotocin-an antibiotic used to induce diabetes on experimental animals. J Educ Health Sport.

[15] Hamza AA, Mohamed MG, Lashin FM, Amin A (2020). Dandelion prevents liver fibrosis, inflammatory response, and oxidative stress in rats. J Basic Appl Zool.

[16] Ashktorab H, Soleimani A, Singh G, Amin A, Tabtabaei S, Latella G (2019). Saffron: the golden spice with therapeutic properties on digestive diseases. Nutrients..

[17] Hamza AA, Lashin FM, Gamel M, Hassanin SO, Abdalla Y, Amin A (2020). Hawthorn herbal preparation from *Crataegus oxyacanth*a attenuates in vivo carbon tetrachloride -induced hepatic fibrosis via modulating oxidative stress and inflammation. Antioxidants (Basel, Switzerland).

[18] Amin A, Farrukh A, Murali C, Soleimani A, Praz F, Graziani G (2021). Saffron and its major ingredients' effect on colon cancer cells with mismatch repair deficiency and microsatellite instability. Molecules (Basel, Switzerland).

[19] Mu C, Sheng Y, Wang Q, Amin A, Li X, Xie Y (2021). Potential compound from herbal food of *Rhizoma Polygonati* for treatment of COVID-19 analysed by network pharmacology: viral and cancer signaling mechanisms. J Funct Foods.

[20] Ismail H, Rasheed A, Haq I-u, Jafri L, Ullah N, Dilshad E (2017). Five indigenous plants of Pakistan with anti-nociceptive, anti-inflammatory, antidepressant, and anticoagulant properties in Sprague Dawley rats. Evid Based Complement Alternat Med.

[21] Harlan JR (1986). Lettuce and the sycomore: sex and romance in ancient Egypt. Econ Bot.

[22] Ismail H, Mirza B (2015). Evaluation of analgesic, anti-inflammatory, anti-depressant and anti-coagulant properties of *Lactuca sativa* (CV. Grand Rapids) plant tissues and cell suspension in rats. BMC Complement Altern Med.

[23] Poddar S, Sarkar T, Choudhury S, Chatterjee S, Ghosh P (2020). Indian traditional medicinal plants: a concise review. Int J Bot Stud.

[24] Adesso S, Pepe G, Sommella E, Manfra M, Scopa A, Sofo A (2016). Anti-inflammatory and anti-oxidant activity of polyphenolic extracts from *Lactuca sativa* (var. Maravilla de Verano) under different farming methods. J Sci Food Agric.

[25] Ismail H, Gillespie AL, Calderwood D, Iqbal H, Gallagher C, Chevallier OP (2019). The health promoting bioactivities of *Lactuca sativa* can be enhanced by genetic modulation of plant secondary metabolites. Metabolites..

[26] Im S-E, Yoon H, Nam T-G, Heo HJ, Lee CY, Kim D-O (2010). Antineurodegenerative effect of phenolic extracts and caffeic acid derivatives in romaine lettuce on neuron-like PC-12 cells. J Med Food.

[27] Okada Y, Okada M (2013). Protective effects of plant seed extracts against amyloid β-induced neurotoxicity in cultured hippocampal neurons. J Pharm Bioallied Sci.

[28] Harsha S, Anilakumar K (2013). Protection against aluminium neurotoxicity: a repertoire of lettuce anti-oxidants. Biomed Aging Pathol.

[29] Malik J, Kaur J, Choudhary S (2018). Standardised extract of *Lactuca sativa* Linn. And its fractions abrogates scopolamine-induced amnesia in mice: a possible cholinergic and anti-oxidant mechanism. Nutr Neurosci.

[30] EPA P (2005). National Environmental Policy 2005. Government of Pakistan, Ministry of Environment.

[31] Ismail H, Dilshad E, Waheed MT, Sajid M, Kayani WK, Mirza B (2016). Transformation of *Lactuca sativa* L. with rol C gene results in increased anti-oxidant potential and enhanced analgesic, anti-inflammatory and antidepressant activities in vivo. 3 Biotech..

[32] Ismail H, Dilshad E, Waheed MT, Mirza B (2017). Transformation of lettuce with rol ABC genes: extracts show enhanced antioxidant, analgesic, anti-inflammatory, antidepressant, and anticoagulant activities in rats. Appl Biochem Biotechnol.

[33] Kayani WK, Dilshad E, Ahmed T, Ismail H, Mirza B (2016). Evaluation of *Ajuga bracteosa* for anti-oxidant, anti-inflammatory, analgesic, antidepressant and anticoagulant activities. BMC Complement Altern Med.

[34] Sajid M, Khan MR, Shah NA, Shah SA, Ismail H, Younis T (2016). Phytochemical, anti-oxidant and hepatoprotective effects of *Alnus nitida* bark in carbon tetrachloride challenged Sprague Dawley rats. BMC Complement Altern Med.

[35] Ismail H, Mirza B, Haq I-u, Shabbir M, Akhter Z, Basharat A (2015). Synthesis, characterisation, and pharmacological evaluation of selected aromatic amines. J Chem.

[36] Dilshad E, Ismail H, Cusido RM, Palazon J, Ramirez-Estrada K, Mirza B (2016). Rol genes enhance the biosynthesis of anti-oxidants in *Artemisia carvifolia* Buch. BMC Plant Biol.

[37] Shabbir M, Akhter Z, Raithby PR, Thomas LH, Ismail H, Arshad F (2017). Synthesis, characterisation and biological properties of novel ON donor bidentate Schiff bases and their copper (II) complexes. J Coord Chem.

[38] Saeed A, Channar PA, Larik FA, Jabeen F, Muqadar U, Saeed S (2017). Design, synthesis, molecular docking studies of organotin-drug derivatives as multi-target agents against antibacterial, antifungal, α-amylase, α-glucosidase and butyrylcholinesterase. Inorg Chim Acta.

[39] Ellman GL, Courtney KD, Andres V, Featherstone RM (1961). A new and rapid colorimetric determination of acetylcholinesterase activity. Biochem Pharmacol.

[40] Mushtaq A, Ali S, Tahir MN, Ismail H, Mirza B, Saadiq M (2017). New bioactive heteroleptic copper (II) carboxylates: structure, enzymatic and DNA-binding studies. Acta Chim Slov.

[41] Larik FA, Saeed A, Channar PA, Ismail H, Dilshad E, Mirza B (2016). New 1-octanoyl-3-aryl thiourea derivatives: solvent-free synthesis, characterisation and multi-target biological activities. Bangladesh J Pharmacol.

[42] Dilshad E, Zafar S, Ismail H, Waheed MT, Cusido RM, Palazon J (2016). Effect of rol genes on polyphenols biosynthesis in *Artemisia annua* and their effect on anti-oxidant and cytotoxic potential of the plant. Appl Biochem Biotechnol.

[43] Kilkenny C, Browne WJ, Cuthill IC, Emerson M, Altman DG (2014). Improving bioscience research reporting: the ARRIVE guidelines for reporting animal research. Animals..

[44] Hashmi WJ, Ismail H, Mehmood F, Mirza B (2018). Neuroprotective, antidiabetic and anti-oxidant effect of *Hedera nepalensis* and lupeol against STZ+ AlCl 3 induced rats model. DARU J Pharm Sci.

[45] OECD. 420-Guidelines for testing of chemicals, acute oral toxicity-fixed dose procedure. Organisation for Economic Cooperation and Development (OECD). Paris: OECD; 2001.

[46] Bradford MM (1976). A rapid and sensitive method for the quantitation of microgram quantities of protein utilising the principle of protein-dye binding. Anal Biochem.

[47] Ohkawa H, Ohishi N, Yagi K (1979). Assay for lipid peroxides in animal tissues by thiobarbituric acid reaction. Anal Biochem.

[48] Aebi H. Catalase in vitro. Methods in Enzymology. 105: Elsevier; 1984. p. 121–6. 10.1016/s0076-6879(84)05016-3 6727660

[49] Ellman. (1959). Tissue sulfhydryl groups. Arch Biochem Biophys.

[50] Bannister JV, Calabrese L. Assays for superoxide dismutase. Methods Biochem Anal. 1987;105:279–312. 10.1002/9780470110539.ch5 3033431

[51] Alici EH, Arabaci G, Determination of SOD, POD. PPO and cat enzyme activities in Rumex obtusifolius L. Annu Res Rev Biol. 2016;11(3):1–7.

[52] Nijveldt RJ, Van Nood E, Van Hoorn DE, Boelens PG, Van Norren K, Van Leeuwen PA (2001). Flavonoids: a review of probable mechanisms of action and potential applications. Am J Clin Nutr.

[53] Prieto P, Pineda M, Aguilar M (1999). Spectrophotometric quantitation of anti-oxidant capacity through the formation of a phosphomolybdenum complex: specific application to the determination of vitamin E. Anal Biochem.

[54] Li X, Wang X, Chen D, Chen S (2011). Antioxidant activity and mechanism of protocatechuic acid in vitro. Funct Foods Health Dis.

[55] Saravanan K, Aradhya SM (2011). Potential nutraceutical food beverage with anti-oxidant properties from banana plant bio-waste (pseudostem and rhizome). Food Funct.

[56] Barku V, Opoku-Boahen Y, Owusu-Ansah E, Mensah E (2013). Antioxidant activity and the estimation of total phenolic and flavonoid contents of the root extract of *Amaranthus spinosus*. Asian J Plant Sci Res.

[57] Gray J (1978). Measurement of lipid oxidation: a review. J Am Oil Chem Soc.

[58] Spandana SS, Nutan SR, Nethravathi B, Giresha AS (2019). In vitro anti-oxidant activities and phytochemical content of aqueous extracts of *Vernonia anthelmintica* (bitter cumin) seed. In Vitro.

[59] Jun T, Liancai Z, Bochu W (2007). Effects of quercetin on DNA damage induced by copper ion. Int J Pharmacol.

[60] Goto Y, Yamada K, Ohyama T, Matsuo T, Odaka H, Ikeda H (1995). An α-glucosidase inhibitor, AO-128, retards carbohydrate absorption in rats and humans. Diabetes Res Clin Pract.

[61] Kumar S, Narwal S, Kumar V, Prakash O (2011). α-Glucosidase inhibitors from plants: a natural approach to treat diabetes. Pharmacogn Rev.

[62] Sales PM, Souza PM, Simeoni LA, Magalhães PO, Silveira D (2012). α-Amylase inhibitors: a review of raw material and isolated compounds from plant source. J Pharm Pharm Sci.

[63] Mentreddy S, Mohamed A, Rimando A (2005). Medicinal plants with hypoglycemic/anti-hyperglycemic properties: a review. Association for the Advancement of Industrial Crops Conference.

[64] Lo Piparo E, Scheib H, Frei N, Williamson G, Grigorov M, Chou CJ (2008). Flavonoids for controlling starch digestion: structural requirements for inhibiting human α-amylase. J Med Chem.

[65] Francis PT, Palmer AM, Snape M, Wilcock GK (1999). The cholinergic hypothesis of Alzheimer's disease: a review of progress. J Neurol Neurosurg Psychiatry.

[66] Pohanka M, Vlček V, Kuča K, Cabal J, Fusek J (2012). Acetylcholinesterase based colorimetric dipsticks for military performance: principles and construction. Adv Mil Technol.

[67] Holzgrabe U, Kapková P, Alptüzün V, Scheiber J, Kugelmann E (2007). Targeting acetylcholinesterase to treat neurodegeneration. Expert Opin Ther Targets.

[68] Darvesh S, Hopkins DA, Geula C (2003). Neurobiology of butyrylcholinesterase. Nat Rev Neurosci.

[69] Ferreira JF, Luthria DL, Sasaki T, Heyerick A (2010). Flavonoids from *Artemisia annua* L. as anti-oxidants and their potential synergism with artemisinin against malaria and cancer. Molecules..

[70] Singh NP, Ferreira JF, Park JS, Lai HC (2011). Cytotoxicity of ethanolic extracts of *Artemisia annua* to Molt-4 human leukemia cells. Planta Med.

[71] Lee TK, Vairappan CS (2011). Anti-oxidant, antibacterial and cytotoxic activities of essential oils and ethanol extracts of selected south east Asian herbs. J Med Plants Res.

[72] Mojarrab M, Jamshidi M, Ahmadi F, Alizadeh E, Hosseinzadeh L (2013). Extracts of *Artemisia ciniformis* protect cytotoxicity induced by hydrogen peroxide in H9c2 cardiac muscle cells through the inhibition of reactive oxygen species. Adv Pharmacol Sci.

[73] Barone P (2010). Neurotransmission in Parkinson's disease: beyond dopamine. Eur J Neurol.

[74] Khan A, Muhamad NA, Ismail H, Nasir A, Khalil AAK, Anwar Y (2020). Potential nutraceutical benefits of in vivo grown saffron (*Crocus sativus* L.) as analgesic, anti-inflammatory, anticoagulant, and antidepressant in mice. Plants..

[75] Chatterjee M, Verma R, Lakshmi V, Sengupta S, Verma AK, Mahdi AA (2013). Anxiolytic effects of *Plumeria rubra* var. acutifolia (Poiret) L. flower extracts in the elevated plus-maze model of anxiety in mice. Asian J Psychiatr.

[76] Chassot JM, Longhini R, Gazarini L, Mello JCP, de Oliveira RMW (2011). Preclinical evaluation of *Trichilia catigua* extracts on the central nervous system of mice. J Ethnopharmacol.

[77] Ismail H, Amanat MA, Iqbal A, Mirza B (2018). Medicinal plants: a complementary and alternative antidepressant therapy. Curr Pharm Des.

[78] Lu C, Wang Y, Xu T, Li Q, Wang D, Zhang L (2018). Genistein ameliorates scopolamine-induced amnesia in mice through the regulation of the cholinergic neurotransmission, anti-oxidant system and the ERK/CREB/BDNF signaling. Front Pharmacol.

[79] Rajput MS, Sarkar PD (2017). Modulation of neuro-inflammatory condition, acetylcholinesterase and anti-oxidant levels by genistein attenuates diabetes associated cognitive decline in mice. Chem Biol Interact.

[80] Vasileva LV, Getova DP, Doncheva ND, Marchev AS, Georgiev MI (2016). Beneficial effect of commercial Rhodiola extract in rats with scopolamine-induced memory impairment on active avoidance. J Ethnopharmacol.

[81] Solati J, Soleimani N (2010). Antihyperglycaemia and antilipidaemic effect of *Ziziphus vulgaris* L on streptozotocin induced diabetic adult male Wistar rats. Acta Diabetol.

[82] Lenzen S (2008). The mechanisms of alloxan-and streptozotocin-induced diabetes. Diabetologia..

[83] Bedoya F, Solano F, Lucas M (1996). N-monomethyl-arginine and nicotinamide prevent streptozotocin-induced double strand DNA break formation in pancreatic rat islets. Experientia..

[84] Adeyemi DO, Ukwenya VO, Obuotor EM, Adewole SO (2014). Anti-hepatotoxic activities of *Hibiscus sabdariffa* L. in animal model of streptozotocin diabetes-induced liver damage. BMC Complement Altern Med.

[85] Kyznetsova MY, Makieieva OM, Lavrovska DO, Tymoshenko MO, Sheverova DP, Halenova TI (2015). Effect of aqueous extract from *Phaseolus vulgaris* pods on lipid peroxidation and anti-oxidant enzymes activity in the liver and kidney of diabetic rats. J Appl Pharm Sci.

[86] Kennedy AL, Lyons TJ (1997). Glycation, oxidation, and lipoxidation in the development of diabetic complications. Metabolism..

[87] Yagi K (1976). A simple fluorometric assay for lipoperoxide in blood plasma. Biochem Med.

[88] Draper H, Squires E, Mahmoodi H, Wu J, Agarwal S, Hadley M (1993). A comparative evaluation of thiobarbituric acid methods for the determination of malondialdehyde in biological materials. Free Radic Biol Med.

[89] RG A (2005). The physiological and biochemical effects of diabetes on the balance between oxidative stress and anti-oxidant defense system. Med J Islamic World Acad Sci.

[90] Otitoju O, Onwurah IN, Otitoju GT, Ugwu CE (2008). Oxidative stress and superoxide dismutase activity in brain of rats fed with diet containing permethrin. Biokemistri..

[91] Rajasekaran S, Sivagnanam K, Subramanian S (2005). Antioxidant effect of *Aloe vera* gel extract in streptozotocin-induced diabetes in rats. Pharmacol Rep.

[92] Chugh S, Kakkar R, Kalra S, Sharma A (1999). An evaluation of oxidative stress in diabetes mellitus during uncontrolled and controlled state and after vitamin E supplementation. J Assoc Physicians India.

[93] Cnubben NH, Rietjens IM, Wortelboer H, van Zanden J, van Bladeren PJ (2001). The interplay of glutathione-related processes in anti-oxidant defense. Environ Toxicol Pharmacol.

[94] Mdawar B, Ghossoub E, Khoury R (2020). Selective serotonin reuptake inhibitors and Alzheimer's disease. Neural Regen Res.

[95] Cirrito JR, Disabato BM, Restivo JL, Verges DK, Goebel WD, Sathyan A (2011). Serotonin signaling is associated with lower amyloid-β levels and plaques in transgenic mice and humans. Proc Natl Acad Sci.

[96] Ramos-Rodriguez JJ, Molina-Gil S, Rey-Brea R, Berrocoso E, Garcia-Alloza M (2013). Specific serotonergic denervation affects tau pathology and cognition without altering senile plaques deposition in APP/PS1 mice. PLoS One.

[97] Iqbal K, Liu F, Gong C-X, Grundke-Iqbal I (2010). Tau in Alzheimer disease and related tauopathies. Curr Alzheimer Res.

